# Partial nephrectomy in multiple mixed epithelial and stromal tumors (MEST)

**DOI:** 10.1016/j.eucr.2024.102731

**Published:** 2024-04-06

**Authors:** Nasser Simforoosh, Mohammad Sajjad Zabihi, Mehdi Dadpour

**Affiliations:** Shahid Beheshti University of Medical Sciences, Shahid Labbafinejad Medical Center, Center of Excellence in Urology, Urology and Nephrology Research Center, Tehran, Iran

**Keywords:** Mixed epithelial and stromal tumor, Kidney neoplasm, Partial nephrectomy

## Abstract

Mixed epithelial and stromal tumor (MEST) of the kidney is not a common diagnosis. This tumor usually mimics solid-cystic malignant renal tumors and most cases are treated surgically. Here, we presented a 35-year-old female with simultaneous two separate large solid-cystic masses with contrast enhancement in lower and upper pole of left kidney which were managed surgically via partial nephrectomy. Microscopic evaluation showed solid-cystic tumor with variably sized cysts lined by flattened to cuboidal epithelium that was compatible with MEST. Follow up evaluation revealed normal parenchymal renal tissue and proper function without any evidence of local recurrence.

## 1 Introduction

Mixed epithelial and stromal tumor (MEST) of the kidney is infrequent,^1^ most recently defined entity and first described by Michal and Syrucek in 1998.^2^ Previously, MESTs have probably been diagnosed as other lesions such as cystic nephroma, mesoblastic nephroma, leiomyoma and cystic partially differentiated nephroblastoma.^3^, ^4^, ^5^ The classic CT appearance of MEST is a well-circumscribed, multiseptate cystic and solid mass with delayed contrast material enhancement.^6^ Microscopically, the spindle cell component ranged in appearance from scar-like fibrous tissue to leiomyoma-like interlacing fascicles; usually there was a mixture of both.^7^ Most patients are symptomatic that potentially related to the renal mass, including hematuria, flank pain, palpable mass, anemia, and acute pyelonephritis.^6^^,^^8^ Diagnosis of MESTS is usually post-operative^9^ and Nephron-sparing surgery (NSS) is performed for most patients.^7^^,^^10^^,^^11^ Here we present a 35-year-old female patient with simultaneous two separate large incidental MEST in upper and lower pole of the left kidney, which were managed by partial nephrectomy.

## 2 Case presentation

**Patient information and medical history:** Our case was a 35-year-old female who had noticed two large solid-cystic lesions in the lower and upper poles of left kidney during an ultrasound checkup due to menstrual problems. She did not have any symptoms such as flank pain, hematuria, anemia and pyelonephritis. All the lab data including creatinine and GFR were within the normal range. No microscopic hematuria or leukocytosis were detected in urine analysis and complete blood cell count, respectively.

**Fig. 1:**
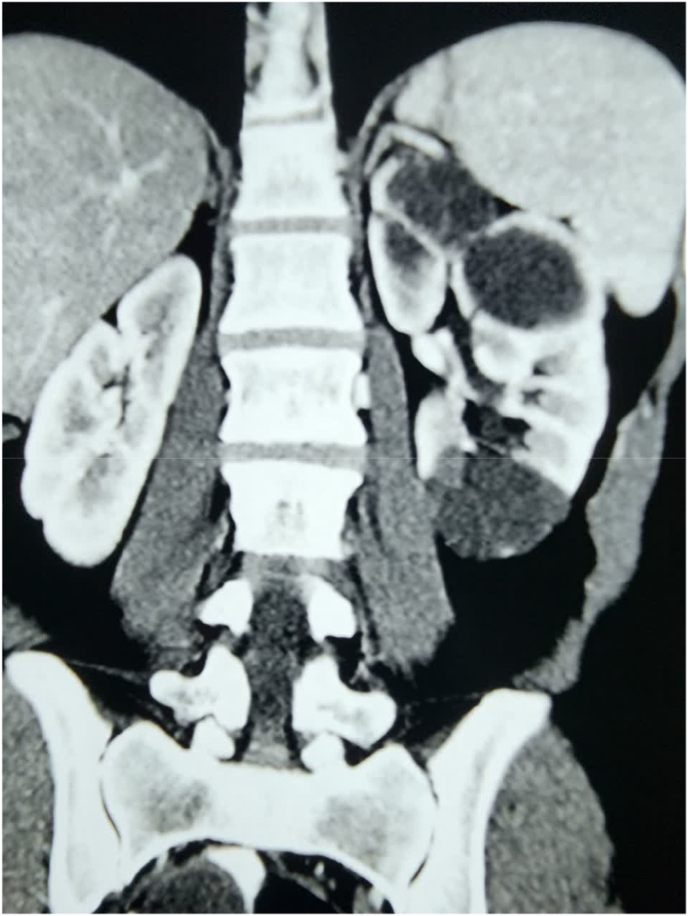
Preoperative CT scan revealed two separate well-circumscribe masses on the upper pole and lower pole of the left kidney with mixed solid and cystic components that contain thick septa with delayed contrast enhancement.

**Fig. 2:**
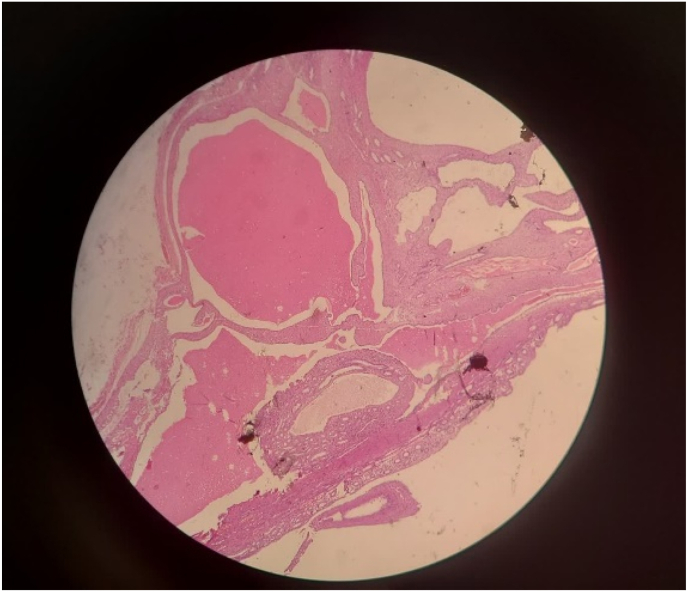
Solid-cystic tumor with variably sized cysts lined by flattened to cuboidal epithelium compatible with Mixed epithelial and stromal tumor.

**Diagnosis and intervention:** CT scan revealed two 3cm and 4cm separate well-circumscribe masses on the upper pole and lower pole of the left kidney with mixed solid and cystic components that contain thick septa with delayed contrast enhancement (Fig. 1). Considering all above, the patient was scheduled for surgical intervention. Under general anesthesia and flank position, the patient underwent open partial nephrectomy. Microscopic evaluation showed that the lesions include cysts lined by cuboidal to flattened columnar epithelium and variable amounts of intervening spindle cell stroma that comprised bland elongated cells with wispy cytoplasm (Fig. 2). All this data plus immune-histo-chemistry evaluation were compatible with mixed epithelial and stromal tumor of the kidney.

**Fig. 3:**
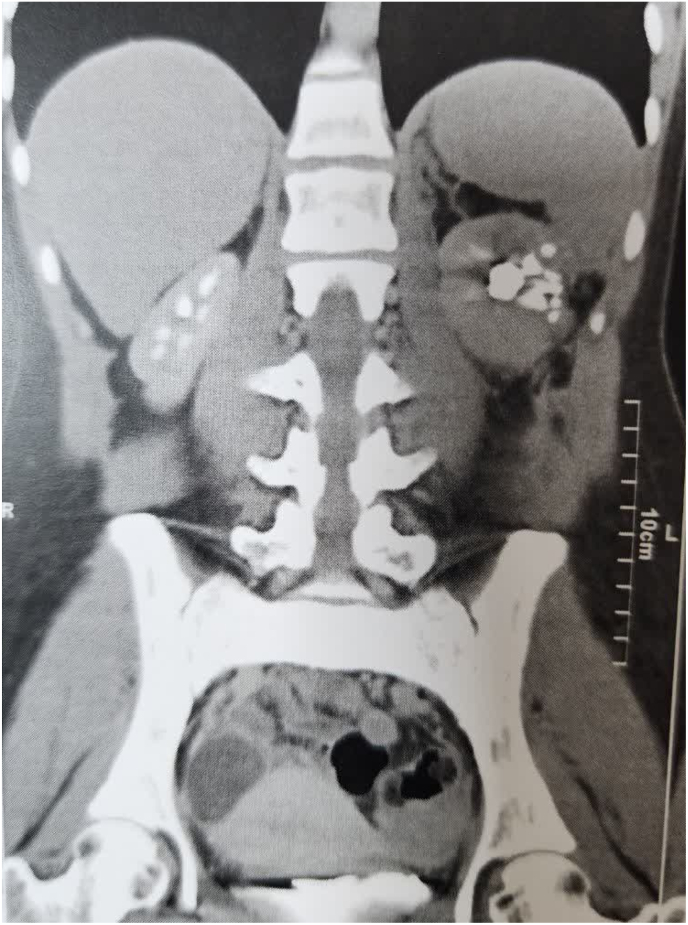
Follow up CT scan with IV contrast revealed normal parenchymal renal tissue and proper function in uro-gram phase without any evidence of local recurrence.

**Follow up:** All the lab data including creatinine and GFR were within the normal range with no change compared to pre-operative measures and the follow up CT scan with IV contrast revealed normal parenchymal renal tissue and proper function in uro-gram phase without any evidence of local recurrence after 6 months follow up (Fig. 3).

## 3 Discussion

Mixed epithelial and stromal tumor is a rare kidney tumor that was first reported in 1998. After that, several reports were published, which helped to enter this pathology of benign kidney tumors into the guidelines. Adsay et al.^12^ reported 12 cases of MEST as a clinicopathologic study in 2000. They emphasized that this entity is a benign tumor and should be distinguished from other renal neoplasms. They hypothesized that the tumor growth may be influenced by female hormones. In 2004, Michal et al.^13^ reported 22 cases of MEST among 8000 patients with renal tumor in their center. The aim of their study was to differentiated MEST from cystic nephromas, cystic partially differentiated nephroblastomas and mesoblastic nephromas. They found MESTK is often macroscopically cystic organized. Microscopic examination shows dimorphism tumor with variable epithelial component cysts that can present with a range of Mullerian phenomena plus a stromal component morphologically and immunohistochemically identical to ovaries. Stroma MESTK represents the renal equivalent of ovarian-like stromal-like cystic tumors Bile duct and pancreas. MESTK behaves benevolent way. It can be assumed that MESTK is caused by a disturbed hormonal environment, because usually occurs in middle-aged and elderly women and many patients have long-term estrogen use. In a large case study, Calio et al.^14^ described the clinicopathologic characteristics of 53 cases of MEST in 2016. They focused on histopathologic tumor features and concluded that the main feature is the complexity and heterogeneity of both the epithelial and stromal units, related to the number of sections submitted and analyzed. Immunohistochemical profiling of the cellular stromal component showed smooth muscle differentiation and repeated progesterone receptor and estrogen receptor positivity. Feng et al.^15^ evaluated 13 cases of MEST from 2016 to 2022 and concluded that there was a high degree of overlap with cystic renal neoplasm, and the rate of missed and misdiagnosis was extremely high.

One of the problems about renal benign tumors is that they usually diagnosed as malignant tumors, pre-operatively and pathologic evaluation confirm their benignity, post-operatively. Most patients with MEST are symptomatically and present with flank pain, hematuria or mass. Radiologic evaluation and CT scans are usually compatible with a well-circumscribed cystic mass with solid components and thick or thin septa with contrast enhancement. So, Preoperative diagnosis of this tumor is difficult, and usually a surgical plan is considered, and Nephron-sparing surgery (NSS) is the best choice. This was the case of a young woman who presented with incidental asymptomatic renal tumors. Simultaneous two completely separate masses in the upper and lower poles of the kidney is not a common finding of MESTs and was special characteristic of this case that promote us to report. Although we did not have information about the benign nature of the tumors, pre-operatively, we performed partial nephrectomy as a nephron sparing surgery for both masses and post-op evaluation revealed proper function of the remaining renal tissue. Pre-operative percutaneous biopsy may help us to diagnose and distinguish from the malignant tumors and renal cell carcinoma, but due to the rarity of this entity, we do not recommend to perform biopsy for all cases with this pre-operative features.

Most MEST lesions are benign and recurrence at the site of operation or metastasis is uncommon, although aggressive and metastatic cases have also been reported in some articles.^16^^,^^17^ In our case, no recurrence of tumor or metastasis has been observed, and the function of the left kidney has been good in 6-months follow-up.

## 4 Conclusion

Here, we presented a 35-year-old female with simultaneous two separate large solid-cystic masses with contrast enhancement in lower pole and upper pole of left kidney which were was compatible with mixed epithelial and stromal tumor of the kidney that were managed surgically via partial nephrectomy.

## CRediT authorship contribution statement

**Nasser Simforoosh:** Conceptualization, Data curation, Investigation, Project administration, Resources, Supervision, Validation, Writing – review & editing. **Mohammad Sajjad Zabihi:** Data curation, Writing – original draft. **Mehdi Dadpour:** Conceptualization, Supervision, Validation, Writing – original draft, Writing – review & editing.
